# Borophene-Based Anisotropic Metamaterial Perfect Absorber for Refractive Index Sensing

**DOI:** 10.3390/nano15070509

**Published:** 2025-03-28

**Authors:** Zichen Lin, Haorui Yang, Gui Jin, Ying Zhu, Bin Tang

**Affiliations:** 1 School of Microelectronics, Changzhou University, Changzhou 213164, China; s22060858030@smail.cczu.edu.cn (Z.L.); s24060809031@smail.cczu.edu.cn (H.Y.); s22060809028@smail.cczu.edu.cn (Y.Z.); 2 Department of Electronic Information and Electronic Engineering, Xiangnan University, Chenzhou 423000, China

**Keywords:** borophene, metamaterial perfect absorber, refractive sensing

## Abstract

Borophene, as a novel two-dimensional (2D) material, has garnered significant interest due to its exceptional optoelectronic properties, including anisotropic plasmonic response high carrier mobility, etc. In this work, we theoretically propose a borophene-based anisotropic metamaterial perfect absorber using the finite-difference time-domain (FDTD) method. The research results show that the proposed metamaterial exhibits triple-band perfect electromagnetic absorption characteristics when the polarization direction of electromagnetic wave is along the zigzag direction of borophene, and the resonant absorption wavelengths can be adjusted by varying the carrier mobility of borophene. Furthermore, as an application of the proposed perfect absorber, we investigate the refractive sensing properties of the borophene-based metamaterial. When the carrier density of borophene is 4.0 × 10^19^ m^−2^, the maximum refractive index sensitivity of the designed absorber is up to 867 nm/RIU, with a figure of merit of 11.71 RIU^−1^, which has promising applications in the field of biochemical sensing and special environmental detection.

## 1. Introduction

Metamaterials are artificially designed composite materials arranged in periodic structures, exhibiting novel electromagnetic characteristics not found in natural materials, such as negative refraction [1], inverse Doppler effect [2], and inverse Cherenkov radiation [3]. Over the past decades, various devices based on metamaterials have been designed to achieve specific functions including sensors [4], beam splitter [5], and polarization converters [6]. In particular, since Landy first proposed the metal-insulator-metal (MIM) metamaterial perfect absorber [7], it has attracted much attention due to high absorption efficiency and lightweight structure [8,9]. Researches on the applications of MIM absorbers are also being carried out extensively, such as radar cloaking [10], infrared detection [11], solar cells [12], color filters [13], mechanical thermal sensors [14], and so on. However, the MIM absorbers usually exhibit fixed resonance frequencies, which are also accompanied by the high Ohmic losses [15,16].

Two-dimensional (2D) materials provide an ideal platform to achieve the active adjustment of work frequency and the tailoring of the absorption bandwidth [17,18]. Therefore, it is desirable to design metamaterial absorber by combining with 2D materials, such as graphene [19,20,21], black phosphorus [22,23] and MoO_3_ [24,25]. Recently, borophene [26,27], as a new emerging 2D material, has been attracting increasing attention due to its unique electrical, optical, and mechanical properties. In contrast to other 2D materials, borophene exhibits a carrier density that is several orders of magnitude higher, thereby earning it the moniker of a 2D metal. This characteristic endows plasmonic devices based on borophene with the capability to operate within the visible and near-infrared wavelength regimes, facilitating their potential applications in various optical and optoelectronic systems [28,29]. Furthermore, borophene has the potential to generate more intriguing physical phenomena due to its asymmetric crystal structures in the armchair (AC) and zigzag (ZZ) directions [30,31]. These characteristics drive further researches on borophene-based metamaterials [32]. Particularly, borophene devices possess unique advantages such as high mode volume and strong light-confinement in 2D resonators in the atomic scale, resulting in high detection sensitivity [33]. For example, Zhang et al. investigated an infrared sensor using an array of borophene ribbons, which can detect the change in refractive index of the surrounding environment [34]. Liu et al. proposed a borophene-based absorption structure, utilized silicon nitride photonic crystals and silver mirrors to achieve controllable anisotropic absorption [35]. Liu et al. achieved single peak perfect absorption of borophene based on the quasi-bound states in the continuum [36]. Yang et al. proposed a dual tunable absorber by tuning the material perturbation of the borophene and the phase-change material [37]. Nevertheless, compared to single-band absorbers, multi-band absorbers offer the potential for frequency-selective detection, thereby reducing environmental interference and improving detection accuracy [38]. To our knowledge, few works have been reported on multi-band borophene-based metamaterial perfect absorbers and the applications at refractive index sensing in the near-infrared region.

In this paper, we present a near-infrared metamaterial perfect absorber based on borophene, which can be used for refractive index sensing. The proposed absorber exhibits diverse absorption properties for different polarization directions on account of the anisotropy of borophene. The research results show that when the polarization direction of electromagnetic wave is along the ZZ direction of borophene, the proposed metamaterial serves as a triple-band perfect absorber. In addition, the absorption peak can be tuned by adjusting the carrier density of borophene. Furthermore, we investigate the refractive index sensing properties of the proposed absorber, which demonstrates the sensitivity with a maximum value of 867 nm/RIU. The designed metamaterial exhibits high sensitivity and tunability, which may have potential applications in biomedical sensing, disease diagnosis, and trace detection of hazardous substances.

## 2. Structure and Methods

**Figure 1:**
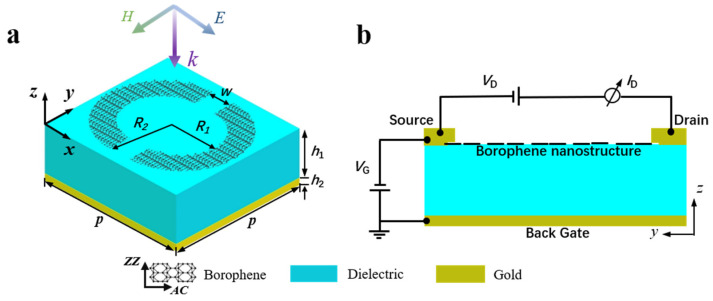
(**a**) Three-dimensional schematic of the proposed metamaterial perfect absorber unit cell. (**b**) The operational schematic of electrical biasing.

**Figure 2:**
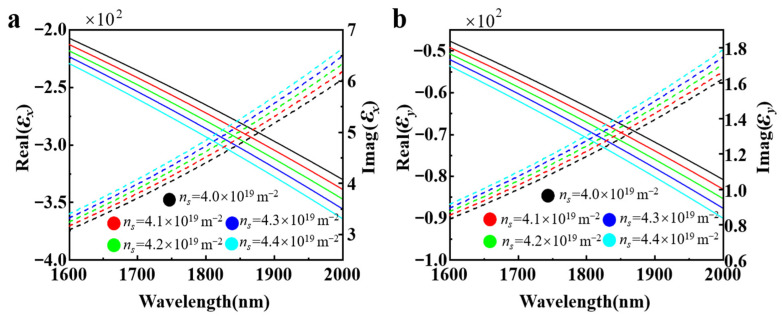
Effective permittivity of the borophene extension along the ZZ direction (**a**) and AC direction (**b**) for different carrier densities. The dashed lines represent the imaginary part, and the solid lines represent the real part.

Figure 1a depicts a three-dimensional structural diagram of the proposed metamaterial unit cell. The unit cell consists of a borophene-based metasurface, a dielectric layer and a gold reflective mirror arranged from top to bottom. The borophene-based metasurface is composed of two borophene semi-rings spaced with a distance of *w* = 40 nm, an inner radius *R*_1_ = 150 nm and an outer radius *R*_2_ = 221 nm. The periods *p* = 500 nm in the x and y directions. The dielectric layer has a thickness of *h*_1_ = 280 nm. The thickness of gold is *h*_2_ = 50 nm, which has reached the penetration depth of gold and can effectively prevent the transmission of electromagnetic waves. In the infrared wavelength regimes, the surface conductivity of the monolayer borophene can be modelled using Drude model as follows [39]:

(1) $$\sigma_{jj}=\frac{iD_{j}}{\pi (\omega +\frac{i}{\tau })},D_{j}=\frac{\pi e^{2}n_{s}}{m_{j}},$$

where $i=\sqrt{-1}$, *j* = *x*, *y* represents the ZZ direction and AC direction, *σ_jj_* is the surface conductivity, *D_j_* is the Drude weight, *m_j_* is the effective electron mass along both directions, and *m_x_* = 1.4 *m*_0_, *m_y_* = 5.2 *m*_0_, where *m*_0_ is the rest mass of electrons. *ω* is the incident light frequency, *τ* is the relaxation time of electron set to 60 fs in this work, *e* and *n_s_* represent electron charge and free carrier density, respectively. The effective permittivity of the borophene can be derived from the surface conductivity along each direction as follows:

(2) $$\varepsilon_{jj}=\varepsilon_{r}+\frac{i\sigma_{jj}}{\varepsilon_{0}\omega d_{B}},$$

where *ε_r_* = 11 is the relative permittivity of borophene, *ε*_0_ is the permittivity of free space, and *d_B_* is the thickness of borophene. Here the thickness of monolayer borophene is chosen as *d_B_* = 0.3 nm, as *d_B_* is sufficiently small compared to the wavelength of interest, enabling the simulations to closely reach the real-world results [26,39]. Figure 2a,b illustrate the effective permittivity of borophene along the AC and ZZ directions for various carrier density, which exhibits obvious anisotropic optical behavior. Therefore, the effective permittivity can be tuned by changing carrier density of borophene, offering the degree of freedom to control the electromagnetic characteristics compared to conventional materials. The carrier density of borophene can be controlled by applying a bias voltage through the electrical back gating method, which can be governed by [40,41]:

(3) $$n_{s}=\frac{C_{\mathrm{gate}}(V_{G}-V_{D})}{e},$$

where *C*_gate_ is the gate oxide capacitance [42], *V*_G_ is the applied gate voltage and *V_D_* is the back-gate-to-source voltage at the Dirac point also known as charge neutral point voltage. The usual method for measuring *V_D_* is to change the gate voltage *V*_G_ and measure the change in current *I_D_* [41,43], and *e* is the charge of the electron. Here, it should be mentioned that the rational values for gate voltage are those that provide a tuneable range of carrier densities compatible with experimental setups. Figure 1b shows the operational schematic of electrical biasing. The optical responses of the proposed metamaterials are simulated via using finite-difference time-domain (FDTD) method. In simulations, the periodic boundary conditions are used in the *x* and *y* directions, and perfectly matching layer boundaries are set in the *z* direction. The plane electromagnetic waves are normally incident on the metamaterial along the negative direction of *z*-axis.

## 3. Results and Discussion

**Figure 3:**
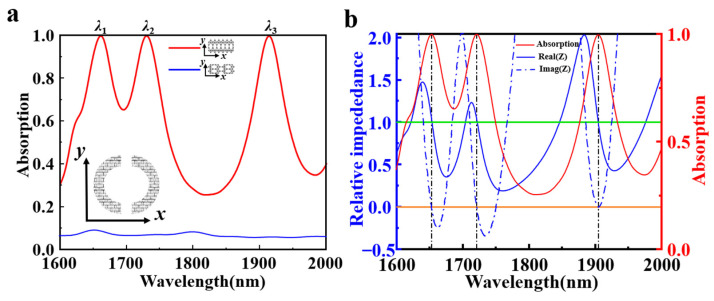
(**a**) Absorption spectra of the designed metamaterial perfect absorber. The red and blue curves denote the ZZ and AC directions of borophene along the direction of *x*-polarization in the legend labeling, respectively. The inset is the top view of patterned borophene unit structure. (**b**) Relative impedance and absorption spectra of the absorber with the ZZ direction of borophene along the direction of polarization.

Figure 3a shows the absorption spectra of the proposed metamaterial with the borophene carrier density *n_s_* = 4.2 × 10^19^ m^−2^. The red and blue curves denote the absorption spectra when the ZZ and AC directions of borophene along the direction of *x*-polarization, respectively. The refractive index of dielectric is set to 1.45, and the permittivity of the gold is referred from Palik [44]. Since the bottom gold layer blocks the transmission of electromagnetic waves, the absorption (*A*) of the metamaterial can be simplified as *A* = 1 − *R*, where “*R*” represents the reflection of the wave. It can be seen from Figure 3a that there exhibit three perfect absorption peaks at the wavelength of *λ*_1_ = 1660 nm, *λ*_2_ = 1729 nm and *λ*_3_ = 1913 nm when the ZZ direction of borophene is along the direction of polarization as shown by the red line. By contrast, there exists a low absorption in the whole wavelength range when the AC direction of borophene is along the direction of polarization. The absorption performance of the metamaterial can be explained by the impedance matching theory. When the equivalent impedance of the metamaterial absorber matches with the impedance of the free space, the absorber can achieve perfect absorption. Absorption and relative impedance satisfy the following relationship [45]:

(4) $$Z=Z_{0}=\sqrt{\frac{\mu_{0}}{\varepsilon_{0}}}\approx 377\Omega ,$$

where *Z* and *Z*_0_ are impedance of the absorber and free space, respectively. *μ*_0_ is the magnetic permeability in free space, *ε*_0_ is the dielectric constant in free space. According to the transmission line theory, the absorptivity can be expressed as:

(5) $$A=1-R=1-{\left|\frac{Z-Z_{0}}{Z+Z_{0}}\right|}^{2}=1-{\left|\frac{Z_{r}-1}{Z_{r}+1}\right|}^{2},$$

where $Z_{r}=Z/Z_{0}$ is the relative impedance. It can be calculated using the following relative impedance formula:

(6) $$Z_{r}=\sqrt{\frac{\mu_{r}(\omega )}{\varepsilon_{r}(\omega )}}=\sqrt{\frac{{\left(1+S_{11}\right)}^{2}+{S_{21}}^{2}}{{\left(1-S_{11}\right)}^{2}-{S_{21}}^{2}}},$$

where *S*_11_ and *S*_21_ are the scattering matrix coefficients reflection and transmission, respectively. According to the Equation (6), the imaginary and real components of the relative impedance are calculated as show in Figure 3b. The relative impedances of the absorber are 1.03 − 0.025i, 1.04 + 0.003i, 0.98 + 0.001i at the resonant wavelengths of *λ*_1_ = 1660 nm, *λ*_2_ = 1729 nm and *λ*_3_ = 1913 nm, separately. Obviously, the real parts of the relative impedances at the three resonant wavelengths are close to 1, and the imaginary parts are approximate to 0. These results indicate that all the relative impedances have a good match with the impedance of the free space, thus resulting in the perfect absorption at these resonant wavelengths.

**Figure 4:**
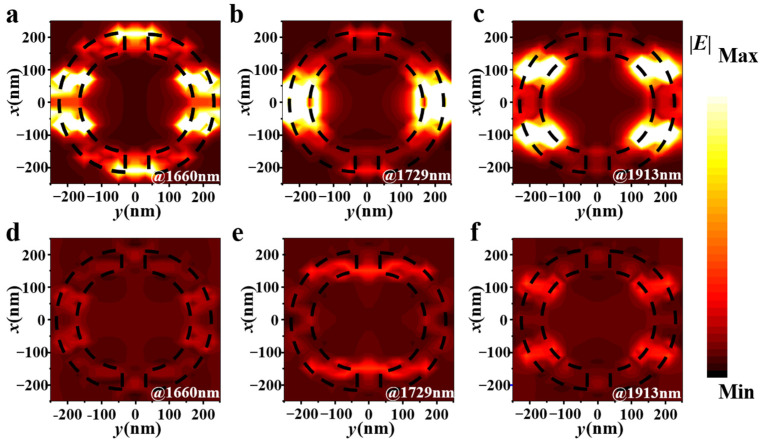
Distributions of electric field at the resonant wavelength *λ*_1_ = 1660 nm, *λ*_2_ = 1729 nm and λ_3_ = 1913 nm at the x-y plane when the direction of polarizations are along ZZ (**a**–**c**) and AC (**d**–**f**) direction of borophene, respectively.

To further investigate the physical mechanism of the three-peak perfect absorption, we provide the distributions of electric fields (|*E*|) at the three resonant wavelengths in the x-y plane, as shown in Figure 4. The black dashed lines sketch the geometry of the patterned borophene metasurface. From Figure 4a–c, it can be observed that when the incident electromagnetic wave is polarized along the ZZ direction of borophene, the electric field distribution varies significantly at the three resonant wavelengths. This variation is caused by the localized surface plasmon resonance (LSPR) effect in the borophene semi-rings. Specifically, it can be found from Figure 4a that there are two resonant excitation modes in each of the borophene semi-ring at the wavelength of 1660 nm. Meanwhile, there appear strongly excited electric fields in the gap edges between two borophene semi-rings. Nevertheless, at the wavelength of 1729 nm, the excited electric field is primarily concentrated in the middle of the borophene semi-ring as shown in Figure 4b. In addition, it can be observed from Figure 4c that at the wavelength of 1913 nm, there exist two resonant excitation modes similar to Figure 4a in each of the borophene semi-ring. These localized surface plasmon resonances can allow the incident electromagnetic waves to be efficiently coupled and interact with the nanostructures. Consequently, the energy of the electromagnetic wave can be accumulated and absorbed in the metasurface, thereby enhancing the absorption efficiency of the electromagnetic wave, and achieving the perfect absorption. By contrast, one can clearly find from Figure 4d–f that the electric field coupling strength is significantly weaker when the polarization direction of the incident electromagnetic wave is along the AC direction of borophene, and the perfect absorption cannot be achieved. Such distinct response phenomena are attributed to the anisotropy induced by the disparities in the effective electron masses and lattice structures of borophene along the two crystal axis directions. These dissimilar atomic structures can generate diverse dielectric responses to the incident electromagnetic waves, thereby resulting in absorption differences.

**Figure 5:**
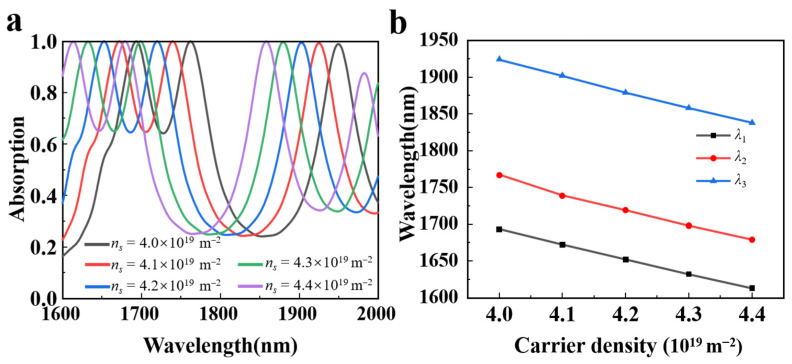
(**a**) Absorption spectra of borophene as a function of carrier density (*n_s_*). (**b**) Wavelength shift of three absorption peaks at different carrier density.

Figure 5a depicts the absorption spectra as the carrier density of borophene is altered. It can be clearly observed that the absorption spectra experience a blue shift as the carrier density *n_s_* increases from 4.0 × 10^19^ m^−2^ to 4.4 × 10^19^ m^−2^, while maintaining a stable perfect absorption. This occurs because, as described in Equation (1), an increase in carrier concentration raises the Drude weight, which decreases the effective dielectric constant and weakens electromagnetic coupling, ultimately shifting the absorption peak to higher frequencies. Concurrently, Figure 5b illustrates the wavelength shift of the three absorption peaks as the carrier density of borophene is adjusted. The results demonstrate that the wavelength shift of the three absorption peaks exhibits a linear change, which allows for the flexible adjustment of the operating wavelength and range of the absorber. This phenomenon can be explained by employing resonant perturbation theory. The absorption peak of the absorber changes due to material perturbation of the borophene, which can be expressed by [46]

(7) $$\begin{array}{c} \frac{\Delta \omega }{\omega_{0}}=\frac{\omega -\omega_{0}}{\omega_{0}}\\ \approx \frac{-{∭}_{v}dV\left[\left(\vec{\Delta \varepsilon }\cdot \vec{E}\right)\cdot \vec{E_{0}^{*}}+(\vec{\Delta \mu }\cdot \vec{H})\cdot \vec{H_{0}^{*}}\right]}{{∭}_{v}dV(\varepsilon {\left|\vec{E_{0}}\right|}^{2}+{\left|\vec{H_{0}}\right|}^{2})}, \end{array}$$

where $\omega_{0}$ denotes the resonant angular frequency, $\overset{⃑}{∆\mu }$ is the change of permeability and $\overset{⃑}{∆\varepsilon }$ is the change of permittivity. Further, *H*, *H*_0_, *E*, and *E*_0_ represent the perturbed magnetic field, undisturbed magnetic field, disturbed electric field, and undisturbed electric field, separately; and $E_{0}^{*}$ and $H_{0}^{*}$ are the complex conjugates of *E*_0_ and *H*_0_, respectively. The denominator of the right-hand side of the equation represents the unperturbed total energy and the numerator represents the change of electromagnetic energy caused by the material perturbation. Therefore, the fractional change in resonance frequency is simply proportional to fractional changes in the total electromagnetic energy. As illustrated in Figure 2, the real and imaginary parts of permittivity decrease with increasing of carrier density, indicating that the change in permittivity ($\overset{⃑}{∆\varepsilon }$) is less than 0. Thus, based on Equation (7), one can conclude that as the carrier density increases, the resonant wavelength shifts toward shorter wavelengths, namely ∆*ω* > 0, demonstrating a blueshift as shown in Figure 5.

**Figure 6:**
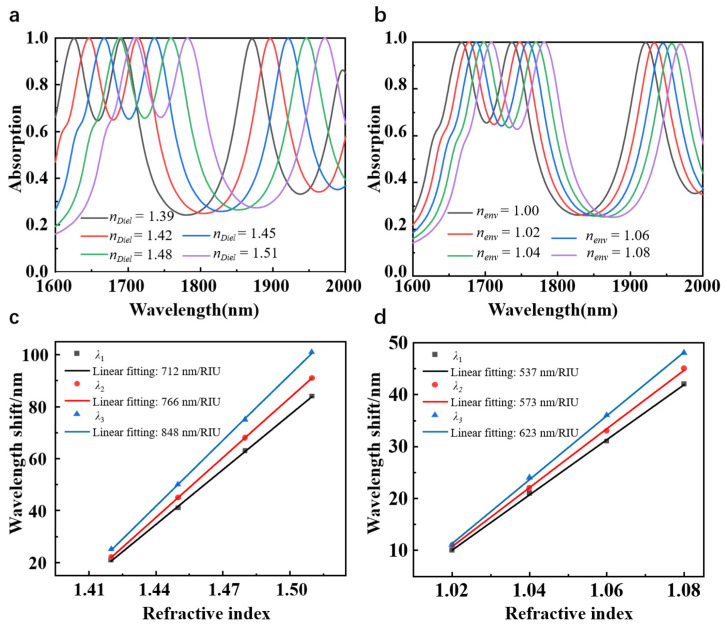
Absorption spectra of absorber as a function wavelength and (**a**) dielectric refractive index, (**b**) environment refractive index. The resonant wavelength shift of the absorber varies with (**c**) the dielectric refractive index and (**d**) the environmental refractive index.

**Figure 7:**
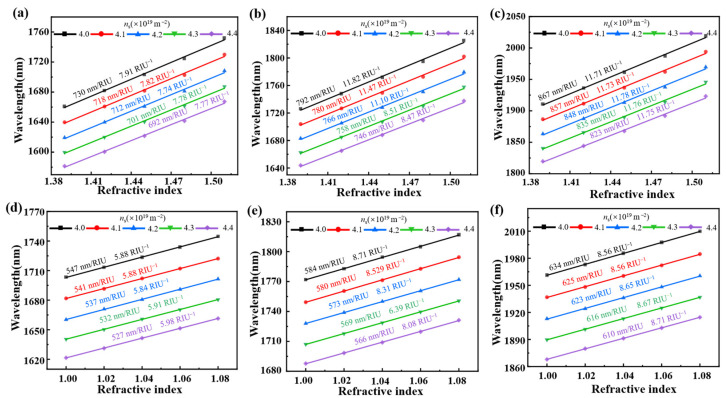
(**a**–**c**) The resonant wavelengths of absorption peak *λ*_1_, *λ*_2_ and *λ*_3_ as functions of *n*_Diel_ under different *n_s_*. (**d**–**f**) The resonant wavelengths of absorption peak *λ*_1_, *λ*_2_ and *λ*_3_ as functions of *n*_env_ under different *n_s_*.

In view of the above characteristics, the proposed borophene-based metamaterial absorber may have practical applications in refractive index sensing. The main advantage of the metamaterial absorber as a sensor lies in its sensitivity to the changes in the resonance wavelengths. Therefore, we further investigate the sensing performance of the proposed metamaterial absorber. Figure 6 calculates the absorption spectra and the corresponding shifts of the resonant wavelengths with the refractive index change of the environment ($n_{env}$) or the dielectric layer ($n_{Diel}$). In Figure 6a, the range for the refractive index of the dielectric layer is chosen between 1.39 and 1.51 and the refractive index of the environment is fixed at 1.00. In Figure 6b, the refractive index of the environment $n_{env}$ is varied from 1.00 to 1.08, while the refractive index of the dielectric layer $n_{Diel}$ is fixed at 1.45. The aforementioned range has been selected as it is capable of detecting of different gases and cells [47,48]. The carrier density is maintained at *n_s_* = 4.2 × 10^19^ m^−2^ throughout the calculations, unless explicitly stated otherwise. In order to have a more accurate and clear understanding of the sensing characteristics of the metamaterial absorber, we introduce two main parameters, namely the sensitivity (*S*) and figure of merit (*FoM*). The sensitivity of the sensor is defined as [49]:

(8) $$S=\frac{\Delta \lambda }{\Delta n},$$

in which ∆n and ∆λ represent the changes of refractive index and resonant wavelength, respectively. Besides, FoM is also an important sensing factor, which is defined as:

(9) $$FoM=\frac{S}{FWHM},$$

where *FWHM* represents the full width at half maximum of the resonance peak. As shown in Figure 6a,b, the absorption spectra of the metamaterial exhibit a red shift as the refractive index $n_{env}$ and $n_{Diel}$ increase, respectively. Figure 6c,d demonstrate the relationship between the relative shifts of three peak wavelengths and different refractive index. It can be observed that the wavelength shifts of the three absorption peaks exhibit disparate linear relationships of change as the refractive index increases. In particular, the change in the peak wavelength *λ*_3_ with the refractive index is the most significant. By contrast, the change in the peak wavelength *λ*_1_ with respect to the refractive index is the smallest among the observed wavelength shifts. In accordance with Equation (8), the sensitivity of the metamaterial as a sensor can be calculated as follows. For the environmental refractive index sensor, the values of sensitivity at the peak wavelengths *λ*_1_, *λ*_2_ and *λ*_3_ reach up to 537 nm/RIU, 573 nm/RIU, and 623 nm/RIU, separately. By contrast, for the dielectric layer refractive index sensor, the values of sensitivity at peak wavelength *λ*_1_ and peak wavelength *λ*_2_ are 712 nm/RIU and 766 nm/RIU, respectively, while the peak wavelength at *λ*_3_ exhibits a higher performance with a value of 848 nm/RIU. In addition, the performance of the sensor can be further evaluated according to Equation (9). Upon calculation, it is found that for the change of environmental refractive index, the *FoM* at *λ*_1_ and *λ*_2_ are 5.84 RIU^−1^ and 8.31 RIU^−1^, while the *FoM* at peak wavelength *λ*_3_ is 8.65 RIU^−1^. By contrast, for the change of dielectric refractive index, the values of *FoM* at *λ*_1_ and *λ*_2_ are 7.74 RIU^−1^ and 11.10 RIU^−1^, respectively, while the value of *FoM* at peak wavelength *λ*_3_ reaches up to 11.78 RIU^−1^. Obviously, it can be observed that the peak wavelength *λ*_3_ provides a more pronounced sensitivity for refractive index detection compared to other absorption peaks. Nevertheless, the presence of other absorption peaks can also facilitate the acquisition of additional detection information, thereby enhancing the accuracy of the detection process and reducing the impact of environmental factors. Figure 7 further reveals the effect of *n_s_* on the sensing performance of each absorption peak. The resonant wavelengths of the three absorption peaks are plotted as functions of *n*_Diel_ and *n*_env_ for sensors with different *n_s_*. As *n*_Diel_ and *n*_env_ increase, all resonant peaks exhibit a redshift. The sensitivity calculated at each resonant peak increases as *n_s_* decreases, demonstrating the highly tunable properties of the borophene-based sensors. Especially, as shown in Figure 7c, when the carrier density of borophene is adjusted to 4.0 × 10^19^ m^−2^, the sensor achieves a maximum refractive index sensitivity of 867 nm/RIU, with a corresponding *FoM* of 11.71 RIU^−1^.

To further demonstrate the innovation of the proposed refractive index sensor based on metamaterial perfect absorber, Table 1 presents a comparative analysis of our proposed metamaterial as a sensor with previous analogous works. As illustrated in Table 1, we enumerate the *S*, *FoM*, and the materials in different works. It can be observed that this work has some advantages compared to other sensors, especially in terms of sensitivity.Sensors designed from silicon exhibit a definite advantage in terms of *FoM*, but their operating wavelength range is limited due to the lower tunability. In contrast, our proposed borophene-based refractive index sensor can adjust the working band by changing the carrier density. These capabilities make our sensor a promising candidate for various applications requiring robust and reliable sensing in complex environments.

**Figure 8:**
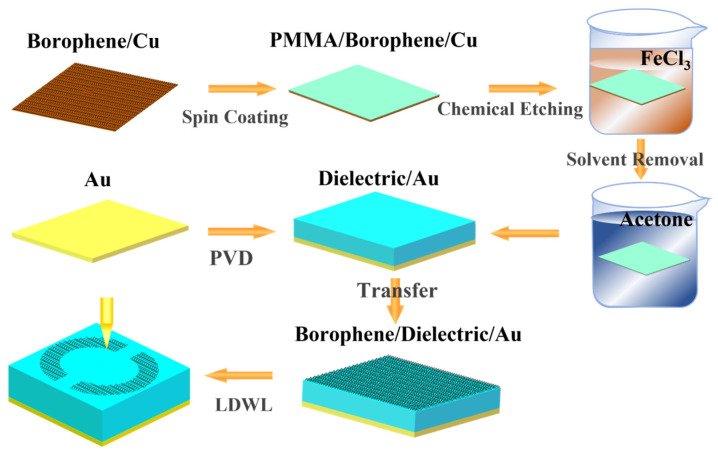
Schematic of the fabrication process of the patterned borophene metamaterials.

Finally, we present a comprehensive schematic of the fabrication process as shown in Figure 8, delineated as follows: (1) Borophene is synthesized on a copper foil via molecular beam epitaxy (MBE) [54]. Prior to its transfer onto the target substrate, the copper foil must be meticulously removed. This is achieved by spin-coating the borophene/copper film with a layer of polymethyl methacrylate (PMMA). Subsequently, the PMMA/borophene/copper assembly is immersed in an FeCl_3_ solution to etch away the underlying copper foil. The resultant borophene/PMMA composite is then subjected to acetone to dissolve and remove the PMMA layer. (2) A dielectric film is deposited onto a gold substrate employing the physical vapor deposition (PVD) technique. (3) The isolated borophene is meticulously transferred and aligned onto the prepared dielectric/Au substrate. Finally, a variety of patterned graphene structures are fabricated using laser direct writing lithography (LDWL).

## 4. Conclusions

In conclusion, we propose a triple-band metamaterial perfect absorber based on borophene. The numerical results show that the absorber exhibits distinct absorption spectra due to the anisotropy of borophene. When the ZZ direction of borophene is along the polarization directions, the proposed metamaterial serves as a triple-band perfect absorber. Meanwhile, the absorption spectra of proposed absorber can be actively adjusted by regulating the carrier density of borophene. Furthermore, we investigate the sensing properties of the proposed absorber as a refractive index sensor. The maximum sensitivity of this sensor can reach up to 867 nm/RIU, with a corresponding *FoM* of 11.71 RIU^−1^. This work offers useful insights for the design of borophene-based nanodevices and has promising applications in the field of biochemical sensing and special environmental detection.

## Figures and Tables

**Table 1:** Performance comparison between the refractive index sensors.

Ref.	*S*(max)	*F* *o* *M*	Material
[30]	561 nm/RIU	5.5	Borophene
[50]	282 nm/RIU	34.3	Graphene
[51]	439 nm/RIU	24.3	Black phosphorus
[52]	405 nm/RIU	2025	Silicon
[53]	61 nm/RIU	0.76	Au
This work	867 nm/RIU	11.71	Borophene

## Data Availability

The data is available from the corresponding author upon reasonable request.
